# A 1-year follow-up study on immunological changes following deep brain stimulation in patients with epilepsy

**DOI:** 10.1038/s41598-021-93265-x

**Published:** 2021-07-02

**Authors:** Pabitra Basnyat, Soila Järvenpää, Jani Raitanen, Marko Pesu, Kai Lehtimäki, Jukka Peltola

**Affiliations:** 1 Department of Neurology, Faculty of Medicine and Health Technology, Tampere University, Arvo Ylpön katu 34, D532, 33520 Tampere, Finland; 2 Department of Neurosciences and Rehabilitation, Tampere University Hospital, Tampere, Finland; 3 Faculty of Social Sciences, Health Sciences, Tampere University, Tampere, Finland; 4 UKK Institute for Health Promotion Research, Tampere, Finland; 5 Immunoregulation, Faculty of Medicine and Health Technology, Tampere University, Tampere, Finland; 6 Fimlab Laboratories, Tampere, Finland

**Keywords:** Biomarkers, Diseases, Health care, Neurology, Translational research, Medical research, Prognostic markers, Immunology, Cytokines, Neuroimmunology, Blood-brain barrier, Cellular neuroscience, Neuroimmunology

## Abstract

The aim of this study was to evaluate the effects of deep brain stimulation of the anterior nucleus of the thalamus (ANT-DBS) on systemic inflammatory responses in patients with drug-resistant epilepsy (DRE). Twenty-two Finnish patients with ANT-DBS implantation were enrolled in this pilot study. Changes in plasma interleukin-6 (IL-6) and interleukin-10 (IL-10) levels were examined using generalized estimating equation models at seven time points (before DBS surgery and 1, 2, 3, 6, 9 and 12 months after implantation). In the whole group, the IL-6/IL-10 ratio decreased significantly over time following ANT-DBS, while the decrease in IL-6 levels and increase in IL-10 levels were not significant. In the responder and nonresponder groups, IL-6 levels remained unchanged during the follow-up. Responders had significantly lower pre-DBS IL-10 levels before the ANT-DBS treatment than nonresponders, but the levels significantly increased over time after the treatment. In addition, responders had a higher pre-DBS IL-6/IL-10 ratio than nonresponders, and the ratio decreased for both groups after treatment, but the decrease did not reach the level of statistical significance. The rate of decrease in the ratio per month tended to be higher in responders than in nonresponders. These results may highlight the anti-inflammatory properties of ANT-DBS treatment associated with its therapeutic effectiveness in patients with DRE. Additional studies are essential to evaluate the potential of the proinflammatory cytokine IL-6, the anti-inflammatory cytokine IL-10, and their ratio as biomarkers to evaluate the therapeutic response to DBS treatment, which could facilitate treatment optimization.

## Introduction

Although a number of antiepileptic drugs (AEDs) are available for the treatment of epilepsy, approximately 30% of patients suffer from drug-resistant epilepsy (DRE)^1^. The surgical treatment options for these patients have included resective surgery and vagus nerve stimulation (VNS). However, many patients still fail to achieve adequate seizure control. Therefore, this group of patients is considered an appropriate candidate for treatment with deep brain stimulation (DBS) therapy. DBS of the anterior nucleus of the thalamus (ANT) is an emerging neuromodulatory therapeutic intervention for DRE that has shown both clinical efficacy and a favourable safety profile^2,3^. The European regulatory authority approved ANT-DBS as an add-on therapy for the treatment of DRE in 2010, while the Food and Drug Administration only approved its use in the United States in April 2018^4^.

The mechanism of action of ANT-DBS in the central nervous system (CNS) is poorly understood. The current hypothesis suggests that electrical stimulation modulates neuronal network excitability and improves or restores neuronal networks that are dysfunctional or pathologically altered, decreasing neuronal cell loss through inhibition of the immune response or modulation of neuronal energy metabolism^5,6^. Several studies have reported the clinically observable effects of DBS; however, studies documenting the effects of ANT-DBS on cellular and molecular profiles are lacking, especially those addressing the systemic inflammatory response. Prognostic markers for the assessment of therapeutic outcomes of ANT-DBS treatment might enable the development of personalized therapeutic interventions and help to avoid the systemic side effects of currently used pharmacological AEDs. ANT-DBS is generally safe and well tolerated, but several adverse events are associated with it, either hardware-related or operation-related complications, which mostly occur around the time of device implantation^3^. While the stimulation-related adverse effect is usually mild and adjustable, a recent report presented a unique case of herpes simplex encephalitis reactivation 1 month after ANT-DBS implantation among patients with DRE, providing evidence for a role of ANT-DBS in the modulation of the inflammatory response^7^.

Based on accumulating evidence, acute and chronic activation of inflammatory pathways are associated with the occurrence of seizures, particularly in patients with drug-resistant focal epilepsy^8^. The role of inflammatory mediators released by brain cells and peripheral immune cells is in the origin of individual seizures and epileptogenesis^9^. In addition, functional interactions between cytokines and neurotransmitters such as glutamate and GABA suggest that these interactions may be responsible for the cytokine-mediated changes in neuronal excitability, stimulating seizure phenomena and the related neuropathology. Epileptic seizures induce the production of a wide spectrum of cytokines that potentially influence the pathogenesis and course of epilepsies. Among them, interleukin (IL)-6, IL-1beta, and IL-1 receptor antagonists are the most commonly studied cytokines in epilepsy due to their potent roles in seizure activity^10^.

In patients with drug-resistant focal epilepsy, especially patients with temporal lobe epilepsy (TLE), the association of proinflammatory cytokines, particularly IL-6, with epileptic disease profiles has been well established^11^. IL-6 is a pro-convulsive and neurotoxic cytokine that increases the production of most acute phase proteins in the liver, such as C-reactive protein, and mediates chronic systemic inflammation in patients with epilepsy^12,13^. Various immune cells produce IL-6, but the most important types are macrophages and monocytes located at sites of inflammation. In our previous studies, we detected chronically increased serum concentrations of IL-6 in patients with TLE compared with those in healthy controls^14^; these patients also exhibited a postictal increase in plasma IL-6 levels^15^. Furthermore, we also observed increased plasma IL-6 levels in patients with TLE compared to patients with extra-TLE, suggesting that the epilepsy type is important for determining seizure-induced IL-6 production^12^. IL-10, an anti-inflammatory and neuroprotective cytokine, is associated with the immunopathology of several diseases, but its role in epilepsy and its anticonvulsant effects are less studied. In the brain, IL-10 is produced by microglia and astrocytes and inhibits the production of proinflammatory cytokines, promotes the production of transforming growth factor-β by astrocytes, promotes neuronal cell survival and regulates adult neurogenesis^16^. Taking into account the broad anti-inflammatory role of IL-10, an animal study revealed the role of IL-10 in the inhibition of IL-1beta production and inflammasome activation in microglia in response to epileptic seizures, suggesting a role for IL-10 in epilepsy treatment^17^. Recently, we detected reduced plasma IL-10 levels in patients with DRE, suggesting the presence of an inadequate systemic anti-inflammatory immune response during chronic epilepsy^18^.

Several studies are available showing VNS-induced immunological changes, particularly longitudinal changes in circulating cytokines in patients with epilepsy^19–21^. However, similar studies of ANT-DBS treatment are lacking. The availability of studies documenting the effects of ANT-DBS on systemic inflammation will help to evaluate the underlying mechanism of action of ANT-DBS. Due to the proconvulsive and anticonvulsive properties of IL-6 and IL-10, respectively, we chose these potent cytokines for this pilot study. We hypothesized that ANT-DBS may induce anti-inflammatory effects on patients with DRE.

## Materials and methods

### Patients

Twenty-two patients with DRE (8 females and 14 males) with a median age of 31.5 years (range 22–56 years) with ANT-DBS implantation were recruited for the current study from Tampere University Hospital. ANT-DBS surgery was performed as reported previously^22^. Patients were considered responders when they presented a ≥ 50% reduction in the seizure frequency between the frequency from 6 months to 1 year after implantation compared to baseline (as measured for at least 3 months prior to the operation). The clinical characteristics of the patients are presented in the online supplement (Supplementary Table S1). The study was approved by the Ethics Committee of the Pirkanmaa Hospital District, Tampere, Finland, and followed the tenets of the Declaration of Helsinki. Written informed consent was obtained from all participants.

### IL-6 and IL-10 measurements

Plasma IL-6 and IL-10 levels were longitudinally measured first at the time of DBS surgery (pre-DBS or 0 months) and at 1, 2, 3, 6, 9 and 12 months after implantation. Depending on the outcome (IL-6, IL-10, or IL-6/IL-10 ratio), each patient had, on average, 6.4 or 6.2 measurements. Based on the response to DBS, patients were classified as (1) responders (n = 9) and (2) nonresponders (n = 13). Plasma IL-6 and IL-10 concentrations were measured using commercially available ELISA kits according to the manufacturer’s protocol (Pelikine Compact, Sanquin, Amsterdam, The Netherlands).

### Statistical analysis

**Figure 1:**
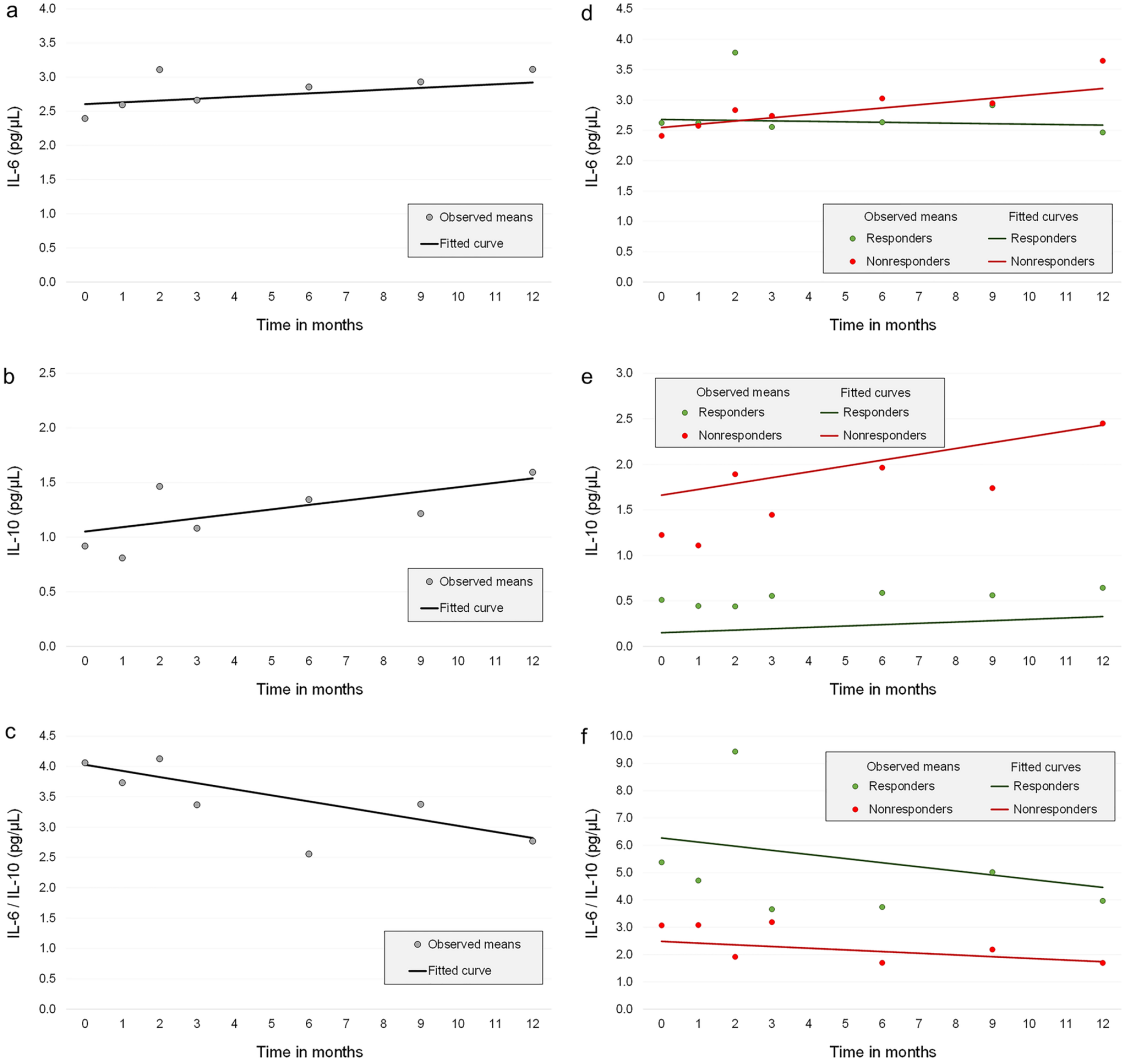
Observed means and fitted curves based on the GEE. Changes in cytokine levels over time in months following deep brain stimulation of the anterior nucleus of the thalamus (ANT-DBS) implantation: (**a**) interleukin-6 (IL-6) levels, (**b**) interleukin-10 (IL-10) levels, (**c**) IL-6/IL-10 ratio in the whole group and (**d**) IL-6 levels, (**e**) IL-10 levels, (**f**) IL-6/IL-10 ratio among groups based on the response to DBS. Time point 0 indicates the time of implantation (baseline or pre-DBS sample). Stata version 16.1 (StataCorp, College Station, Texas, USA) was used to prepare scatterplots and Excel (Microsoft Excel for Microsoft 365 MSO) was used to draw this figure.

Changes in IL-6 levels, IL-10 levels, and the IL-6/IL-10 ratio over time (months) were analysed using a semiparametric generalized estimating equation (GEE) model with robust standard errors in Stata version 16.1 (StataCorp, College Station, Texas, USA). Estimates with their 95% confidence intervals (CIs) were calculated. All the analyses reported were adjusted for age and sex. We also calculated standardized values (z scores) for each outcome to detect potential outliers. Observations with z scores between − 3.29 and + 3.29 were included in the analyses. Graphic representations of the results in Fig. 1 include observed means of the outcomes at each time point and fitted average trajectories based on GEE models adjusted for age and sex. The p-values were considered significant at ≤ 0.05.

### Ethics approval and consent to participate

The Ethics Committee of Tampere University Hospital approved the study (Ethics certification no. R02135) and all participants gave written informed consent.

## Results

### Changes in IL-6 and IL-10 levels in the whole group

When the change in cytokine levels was examined over time after DBS implantation, IL-6 levels remained unchanged (estimate 0.026, 95% CI − 0.015 to 0.068, p = 0.213, Fig. 1a), while IL-10 levels increased by 0.041 pg/mL per month, but the change was close to statistically significant (estimate 0.041, 95% CI − 0.001 to 0.082, p = 0.056, Fig. 1b). The IL-6/IL-10 ratio decreased significantly by 0.101 per month (estimate − 0.101, 95% CI − 0.188 to − 0.013, p = 0.025, Fig. 1c).

### IL-6 and IL-10 levels among responders and nonresponders

#### Pre-DBS

Pre-DBS IL-6 levels did not differ between responders and nonresponders, whereas IL-10 levels were significantly lower in responders than in nonresponders (estimate − 1.511, 95% CI − 2.269 to − 0.753, p < 0.001, Fig. 1e). The pre-DBS IL-6/IL-10 ratio was significantly higher in responders than in nonresponders (estimate 3.789, 95% CI 0.350–7.277, p = 0.031, Fig. 1f).

### Change over time

#### IL-6

The change in IL-6 levels over time was not significant in either responders or nonresponders. IL-6 levels decreased by an average 0.008 pg/mL per month in responders (estimate − 0.008, 95% CI − 0.035 to 0.020, p = 0.581, Fig. 1d), but increased by an average 0.053 pg/mL per month in nonresponders (estimate 0.053, 95% CI 0.013–0.120, p = 0.116). The difference in the change between the two groups was not significant (estimate − 0.061, 95% CI − 0.133 to 0.011, p = 0.096).

#### IL-10

The change in IL-10 levels over time was significant in responders; its value increased on average 0.015 pg/mL per month (estimate 0.015, 95% CI 0.009–0.021, p < 0.001, Fig. 1e). The increase in IL-10 levels in nonresponders was not significant (estimate − 0.064, 95% CI − 0.010 to 0.138, p < 0.090). The difference in the change between the two groups was not significant (estimate − 0.049, 95% CI − 0.124 to 0.025, p = 0.194).

#### IL-6/IL-10 ratio

The IL-6/IL-10 ratio decreased in both groups, but the decrease showed a trend towards statistical significance only in responders. The ratio decreased by an average of 0.151 units per month in responders (estimate − 0.151, 95% CI -0.322 to 0.020, p = 0.084, Fig. 1f) and 0.062 units per month in nonresponders (estimate − 0.062, 95% CI − 0.136 to 0.012, p = 0.103). The IL-6/IL-10 ratio decreased, although not significantly, by an average of 0.089 units more per month in responders than in nonresponders (estimate − 0.089, 95% CI − 0.276 to 0.098, p = 0.352).

The results obtained from the GEE models are reported in Tables 1 and 2. We have also presented the results obtained from the parametric generalized linear mixed models in the online supplement (Supplementary Tables S3–S4). We performed additional analyses using an unadjusted model and a model adjusted for age and sex of IL-6 levels, IL-10 levels, and the IL-6/IL-10 ratio according to the response to treatment with the total number of seizures, time and their interaction serving as covariates. Both unadjusted and adjusted models showed that the interaction between total seizures and time was significant only for the IL-6/IL-10 ratio (p = 0.012) but not for IL-6 (p = 0.388) and IL-10 (p = 0.166) (Supplementary Table S2). Moreover, we have supplemented a panel of line graphs on a continuous scale for individual patients showing the changes in frequency of total seizures in relation to IL-6 levels, IL-10 levels and their ratio over time following ANT-DBS treatment (Supplementary Figs. S1–S3).

**Table 1:** Change in IL-6 and IL-10 levels in the whole cohort.

	Unadjusted			Adjusted for age and sex		
	n	Estimate (95% CI)	p-value	n	Estimate (95% CI)	p-value
**IL-6**						
Change over time	141	0.026 (− 0.015 to 0.068)	0.213	141	0.026 (− 0.015 to 0.068)	0.213
**IL-10**						
Change over time	137	0.041 (− 0.001 to 0.083)	0.056	137	0.041 (− 0.001 to 0.082)	0.056
**IL-6/IL-10**						
Change over time	136	− 0.101 (− 0.189 to − 0.013)	0.025	136	− 0.101 (− 0.188 to − 0.013)	0.025

Estimates with their 95% confidence intervals (CIs) and p values from the generalized estimating equation (GEE) models.

*IL-6* interleukin-6, *IL-10* interleukin-10, *n* number of observations.

**Table 2:** Change in IL-6 and IL-10 levels among responders and nonresponders.

	Unadjusted			Adjusted for age and sex		
	n	Estimate (95% CI)	p value	n	Estimate (95% CI)	p value
**IL-6**	141			141		
Resp vs. nonresp at baseline		0.096 (− 1.915 to 2.108)	0.925		0.133 (− 1.939 to 2.204)	0.900
Change over time, resp		− 0.008 (− 0.035 to 0.020)	0.581		− 0.008 (− 0.035 to 0.020)	0.581
Change over time, nonresp		0.053 (− 0.013 to 0.120)	0.116		0.053 (− 0.013 to 0.120)	0.116
Difference in the change between groups		− 0.061 (− 0.133 to 0.011)	0.096		− 0.061 (− 0.133 to 0.011)	0.096
**IL-10**	137			137		
Resp vs. nonresp at baseline		− 0.964 (− 1.653 to − 0.274)	0.006		− 1.511 (− 2.269 to − 0.753)	< 0.001
Change over time, resp		0.015 (0.015 to 0.009)	< 0.001		0.015 (0.009 to 0.021)	< 0.001
Change over time, nonresp		0.065 (− 0.010 to 0.140)	0.092		0.064 (− 0.010 to 0.138)	0.090
Difference in the change between groups		− 0.050 (− 0.126 to 0.026)	0.195		− 0.049 (− 0.124 to 0.025)	0.194
**IL-6/IL-10**	136			136		
Resp vs. nonresp at baseline		2.913 (− 0.543 to 6.369)	0.099		3.789 (0.350 to 7.277)	0.031
Change over time, resp		− 0.151 (− 0.322 to 0.020)	0.083		− 0.151 (− 0.322 to 0.020)	0.084
Change over time, nonresp		− 0.063 (− 0.137 to 0.010)	0.091		− 0.062 (− 0.136 to 0.012)	0.103
Difference in the change between groups		− 0.088 (− 0.274 to 0.098)	0.356		− 0.089 (− 0.276 to 0.098)	0.352

Estimates with their 95% CIs and p-values from the GEE models.

*IL-6* interleukin-6, *IL-10* interleukin-10, *n* number of observations.

## Discussion

The results of the present study revealed a significant increase in the plasma IL-10 levels over time and unchanged IL-6 levels, while the IL-6/IL-10 ratio decreased in the whole group. In the responder and nonresponder groups, IL-6 levels were unchanged in both groups, but responders exhibited increased IL-10 levels over time following ANT-DBS treatment.

The relationship between neuroinflammation and epilepsy has been widely studied in clinical and experimental studies^23^. We have reported seizure-induced increases in circulating levels of IL-6 in patients with epilepsy in previous studies^12,24^, indicating the excessive production of proinflammatory cytokines by immune cells as a result of neuroinflammation and downstream inflammatory mediators^10^. Although we did not observe a significant difference in IL-6 levels at baseline, the IL-6 levels tended to decrease during follow-up in patients who were responders to ANT-DBS compared to nonresponders. Considering that the higher levels of IL-6 in nonresponders may result from the high seizure frequency, we did not identify any correlation between IL-6 and total seizures of the patients during follow-up (data not shown), indicating that this finding is not a mere epiphenomenon of seizure activity. A longer follow-up study is needed to determine whether ANT-DBS diminishes the systemic inflammatory effects induced by IL-6.

Notably, we observed lower pre-DBS IL-10 levels in responders than in nonresponders. When we investigated whether the different types of epilepsy are responsible for this lower IL-10 level, we did not find any association between baseline IL-10 levels and the epilepsy types. Only one of nine responder patients had TLE, and the remaining had extra TLE or multifocal epilepsy, moreover, only two of 13 nonresponder patients had TLE. Considering the proinflammatory immune status as a principal component in TLE^10,14^, lower pre-DBS IL-10 levels in responders are unlikely to be a result of the epilepsy type, but the finding rather suggests the presence of an inadequate systemic anti-inflammatory immune response as one of the pathophysiological characteristics of chronic epilepsy^18^.

Interestingly, IL-10 levels were increased significantly over time after ANT-DBS treatment in the whole group and the group of responders. These results may highlight the anti-convulsant effects of ANT-DBS on counteracting the proconvulsant effect driven by IL-6. In response to the production of proinflammatory cytokines, anti-inflammatory cytokines such as IL-10, IL-4, IL-13 and TGF-β are produced by activated CNS cells such as microglia and astrocytes secondarily in a delayed manner to limit neurotoxic responses and exert neuroprotective effects^25^. This result may be one of the reasons why IL-10 levels did not display a sharp increase over time in responders, thus signifying the need for a longer follow-up study.

A paucity of clinical reports on ANT-DBS-induced immunological effects on patients with refractory epilepsy exists. However, consistent with our findings, a similar study of VNS-treated patients with DRE reported that treatment induced a reduction in plasma IL-6 levels and an increase in IL-10 levels in responders compared to the control group. In contrast, nonresponders displayed increased plasma IL-6 levels and decreased IL-10 level^21^. Although this clinical report is the first in humans evaluating the immunological response to DBS, other experimental epilepsy studies in rodents have documented the anti-inflammatory and antiapoptotic effects of ANT-DBS^26^, as well as its effects on reducing the proinflammatory state and neuronal injury^27^.

Clinically, DBS treatment reduces short- and long-term seizure frequencies and significantly improves the quality of life of patients; however, the neurobiological concept of DBS-mediated neuroprotective effects remains elusive. Experimental studies have revealed some underlying mechanisms responsible for the anticonvulsant effects of ANT stimulation on epileptic rats^28,29^. In animal studies, ANT-DBS exerted protective effects on hippocampal neurons by enhancing the regeneration of neuronal fibres^30^ and favourably modulating the levels of neurotransmitters in epileptic monkeys^31^. Dysfunction or alteration of the blood–brain barrier (BBB) due to neuroinflammation is a pathogenic hallmark of drug-resistant epilepsy in humans^8^, and ANT-DBS was shown to exert a beneficial effect on reducing BBB dysfunction and promoting anti-inflammatory effects^32^. In addition, alterations in circulatory levels of proinflammatory cytokines such as interleukin-1β, tumor necrosis factor-α (TNF-α), IL-6, and interferon-γ have been reported in an animal model of epilepsy following DBS^33^. Although our results are consistent with previous experimental findings of the beneficial effects of DBS, this preliminary study does not allow us to confirm whether the increased IL-10 levels in responders exert anticonvulsant effects because of the short follow-up period, but our results may reflect the therapeutic effect of ANT-DBS on shifting the immune status from neurotoxic to neuroprotective.

We observed a significantly greater pre-DBS IL-6/IL-10 ratio in responders than in nonresponders, and the ratio decreased over time during follow-up when analysed in the whole group. Responders also presented a decreased IL-6/IL-10 ratio over time, but the change showed only a trend towards statistical significance. The monthly reduction rate was higher in responders than in non-responders, suggesting that this ratio could be employed as a marker of therapeutic response to ANT-DBS. However, this finding should be interpreted carefully considering the effect of total numbers of seizures on the IL-6/IL-10 ratio with time.

The small number of patients is one of the limitations of our study, although the number of observations was approximately 140 in each model; therefore, the findings must be interpreted cautiously. On the other hand, the study group was large compared to previous ANT-DBS clinical studies. Additionally, cytokine measurements were performed only for a single year. DBS might exert variable effects on cytokine production, considering the short-term and long-term effects of the treatment. In our cohort, six patients responded to DBS after 12 months. One patient became a responder at 24 months, three patients at 36 months, one patient at 48 months, and one at 60 months (supplementary Table S1). These six patients were included as nonresponders in the current analysis because we had measured cytokines only for up to 12 months. Therefore, longer follow-up is needed to observe the prolonged effects of DBS on cytokine levels and validate these findings.

Although comparing the ANT-DBS-induced changes in cytokine levels to VNS-induced changes is not the primary aim of this study, existing reports of a VNS-induced reduction in systemic levels of the proinflammatory cytokines IL-6 and TNF-α and increased levels of the anti-inflammatory cytokines IL-10 and transforming growth factor beta (TGF-β)^19,21^ are similar to those in our patients treated with DBS, indicating commonalities in the immunological response elicited by both VNS and DBS. We have previously suggested a putative association between the clinical responses to DBS and prior VNS in patients with DRE^34^.

Taken together, our results highlight the anti-inflammatory properties of ANT-DBS treatment, which may be associated with its therapeutic effectiveness in patients with DRE. While this preliminary report has documented the changes in IL-6 and IL-10 levels and their ratio over time in response to ANT-DBS, additional studies are essential to evaluate the potential of these cytokines to serve as biomarkers of the therapeutic response to ANT-DBS treatment, which may facilitate treatment optimization.

## Supplementary Information

Supplementary Information.

## Data Availability

The data in the current study are available on reasonable request from the corresponding author.
