# Supplementary material for: A 1-year follow-up study on immunological changes following deep brain stimulation in patients with epilepsy
Source: Sci Rep. 2021 Jul 2;11:13765. doi: 10.1038/s41598-021-93265-x (PMC8253825; doi:10.1038/s41598-021-93265-x)

A one-year follow-up study on immunological changes following deep brain stimulation in patients with epilepsy

Pabitra Basnyat[1*], Soila Järvenpää[2], Jani Raitanen[3], Marko Pesu[4], Kai Lehtimäki[2], and Jukka Peltola[1,2]

1. Faculty of Medicine and Health Technology, Tampere University, Tampere, Finland
2. Department of Neurosciences and Rehabilitation, Tampere University Hospital, Tampere, Finland
3. Faculty of Social Sciences, Health Sciences, Tampere University, Tampere, Finland; UKK Institute for Health Promotion Research, Tampere, Finland
4. Immunoregulation, Faculty of Medicine and Health Technology, Tampere University, Tampere, Finland; Fimlab laboratories, Tampere, Finland

Corresponding author

Pabitra Basnyat, PhD

Tampere University

Department of Neurology, Faculty of Medicine and Heath Technology,

Arvo Ylpön katu 34, D532

33520, Tampere, Finland

Tel: +358 442069599

Email : pabitra.basnyat@tuni.fi

Supplementary Table S1. Clinical characteristics of patients.

Patient	Sex	Age (years)	Epilepsy duration (years)	AED baseline → 1 year	Responders status upto 12 months	Etiology	Epileptic zone
1	m	29	9	CBZ 100, CLB 10→20	Responders	Cortical dysplasia	Multifocal
2[d]	f	32	31	CLN 8→6, PHT 200	Non-responders	Cortical dysplasia	Left frontal
3	m	30	18	CBZ 1200, CLB 30	Responders	Cortical dysplasia	Multifocal
4	f	26	19	OXC 1500→1800, CLB 15, ZNS 400→300	Responders	Cortical dysplasia	Multifocal
5	m	22	10	VPA 2500	Non-responders	Encephalitis	Multifocal
6	f	36	10	CBZ 800, LCM 200, LEV 1000, ZNS 400	Responders	Cortical dysplasia	Right frontal
7	m	23	14	CLB 20, LTG 150, VPA 1500, ZNS 400	Responders	Encephalitis	Multifocal
8	m	48	37	CLB 20→40, LCM 400, OXC 1500	Non-responders	Cortical dysplasia	Right temporal
9[c]	f	31	3	CLB 20, LCM 0→500, OXC 1000, ZNS 500→200	Non-responders	Encephalitis	Multifocal
10	m	47	38	CBZ 400, CLB 15, ZNS 400	Responders	Unknown	Frontal

11[b]	f	24	7	CLB 20, OXC 1500, TPR 400	Non-responders	Encephalitis	Multifocal
12	m	24	5	CLB 30, LEV 2000, OXC 1800, TPR 600	Responders	Unknown	Left parietal
13	m	45	9	LCM 400, LEV 3000, OXC 1200	Non-responders	Unknown	Right frontal
14	m	49	44	CBZ 600, LCM 400	Responders	Encephalitis	Right temporal
15[a]	m	40	32	CLB 20→25, OXC 1650, ZNS 400	Non-responders	Cortical dysplasia	Multifocal
16[b]	m	56	42	OXC 1800	Non-responders	Unknown	Multifocal
17	m	29	20	OXC 1800, VPA 1300, ZNS 200	Responders	Unknown	Right frontal
18[b]	m	50	49	CLB 10, LCM 400, PER 8	Non-responders	Ischemic lesion	Right frontal
19	m	22	7	LCM 200, OXC 900, ZNS 500	Non-responders	Encephalitis	Left hemisphere
20	f	54	35	LCM 500→600, TPR 400→300	Non-responders	Hippocampal sclerosis + cortical dysplasia	Left temporal
21	f	48	33	ESL 1600→0, LCM 0→400, PGB 600, ZNS 400	Non-responders	Unknown	Left occipital

22	f	31	8	ESL 2000, LEV 3000, ZNS 400	Non-responders	Encephalitis	Multifocal

Abbreviations: AED, antiepileptic drug; CBZ, carbamazepine; CLB, clobazam; CLN, clonazepam; ESL, eslicarbazepine acetate; LCM, lacosamide; LEV, levetiracetam; LTG, lamotrigine; OXC, oxcarbazepine; PER, perampanel; PGB, pregabalin; PHT, phenytoin; TPR, topiramate; VPA, valproic acid; ZNS, zonisamide; baseline, mean seizure frequency before deep brain stimulator (DBS) was implanted and only AEDs were used to treat epilepsy.

[a]Responder at 24 months (n=1)

[b]Responders at 36 months (n=3)

[c]Responder at 48 months (n=1)

[d]Responder at 60 months (n=1)

Supplementary Table S2. Interaction between total seizures and time for IL-6, IL-10, and IL-6/IL-10 ratio. Cytokines and their ratio as a response and total number of seizures, time and their interaction as covariates. Both unadjusted and adjusted model for age and sex showed that the interaction between total seizures and time is significant for IL-6/IL-10 ratio. Estimates with their 95% confidence intervals (CIs) and p-values from the generalized estimating equation models.

	Unadjusted<br>Estimate (95% CI)	p-value	Adjusted for age and sex<br>Estimate (95% CI)	p-value
IL-6				
Total seizures	0.001 (-0.001 to 0.002)	0.421	0.001 (-0.001 to 0.002)	0.407
Time	0.035 (-0.013 to 0.084)	0.155	0.035 (-0.013 to 0.084)	0.154
Total seizures * Time	-0.0002 (-0.001 to 0.0002)	0.390	-0.0002 (-0.001 to 0.0002)	0.388
Age			0.033 (-0.082 to 0.149)	0.572
Gender			-0.611 (-2.444 to 1.223)	0.514
IL-10				
Total seizures	-0.001 (-0.004 to 0.001)	0.233	-0.001 (-0.004 to 0.001)	0.355
Time	0.024 (-0.023 to 0.071)	0.313	0.024 (-0.023 to 0.071)	0.313
Total seizures * Time	0.0003 (-0.0001 to 0.0008)	0.169	0.0003 (-0.0001 to 0.0008)	0.166
Age			-0.034 (-0.086 to 0.018)	0.200
Gender			-0.462 (-1.402 to 0.477)	0.335
IL-6 / IL-10				
Total seizures	0.011 (0.002 to 0.019)	0.012	0.010 (0.002 to 0.019)	0.016
Time	-0.024 (-0.063 to 0.015)	0.233	-0.024 (-0.063 to 0.016)	0.242
Total seizures * Time	-0.002 (-0.003 to -0.0003)	0.012	-0.002 (-0.003 to -0.0003)	0.012
Age			0.030 (-0.076 to 0.135)	0.580
Gender			0.686 (-2.419 to 3.791)	0.665

IL-6, interleukin-6; IL-10, interleukin-10; n, number of observations.

Supplementary Table S3. Change in IL-6 and IL-10 levels in the whole cohort. Estimates with their 95% confidence intervals (CI) and p-values from the **linear mixed-effects models**.

		Unadjusted			Adjusted for age and sex	
	n	Estimate (95% CI)	p-value	n	Estimate (95% CI)	p-value
IL-6						
Change over time	141	0.026 (-0.013 to 0.065)	0.191	141	0.026 (-0.013 to 0.065)	0.194
IL-10						
Change over time	137	0.045 (0.004 to 0.086)	0.033	137	0.045 (0.004 to 0.087)	0.034
IL-6 / IL-10						
Change over time	136	-0.102 (-0.188 to 0.016)	0.020	136	-0.103 (-0.189 to -0.017)	0.018

IL-6, interleukin-6; IL-10, interleukin-10; n, number of observations.

Supplementary Table S4. Change in IL-6 and IL-10 levels among responders and non-responders. Estimates with their 95% confidence intervals (CI) and p-values from the **linear mixed-effect models.**

		Unadjusted			Adjusted for age and sex	
	n	Estimate (95% CI)	p-value	n	Estimate (95% CI)	p-value
IL-6	141			141		
Resp vs. non-resp at baseline		0.091 (-1.913 to 2.094)	0.929		0.087 (-1.989 to 2.162)	0.935
Change over time, resp		-0.007 (-0.034 to 0.019)	0.586		-0.007 (-0.034 to 0.019)	0.585
Change over time, non-resp		0.052 (-0.010 to 0.113)	0.102		0.051 (-0.011 to 0.113)	0.105
Difference in change between the groups		-0.059 (-0.126 to 0.008)	0.086		-0.059 (-0.126 to 0.009)	0.088
IL-10	137			137		
Resp vs. non-resp at baseline		-0.944 (-1.602 to -0.285)	0.005		-1.339 (-2.021 to -0.657)	<0.001
Change over time, resp		0.015 (0.009 to 0.021)	<0.001		0.015 (0.009 to 0.021)	<0.001
Change over time, non-resp		0.066 (-0.006 to 0.138)	0.071		0.066 (-0.006 to 0.138)	0.072
Difference in change between the groups		-0.051 (-0.123 to 0.021)	0.164		-0.051 (-0.123 to 0.021)	0.164
IL-6 / IL-10	136			136		
Resp vs. non-resp at baseline		2.945 (-0.533 to 6.424)	0.097		3.267 (-0.307 to 6.842)	0.073
Change over time, resp		-0.152 (-0.320 to 0.016)	0.076		-0.152 (-0.320 to 0.016)	0.075
Change over time, non-resp		-0.070 (-0.148 to 0.001)	0.075		-0.071 (-0.149 to 0.008)	0.076
Difference in change between the groups		-0.081 (-0.266 to 0.104)	0.388		-0.082 (-0.267 to 0.103)	0.388

IL-6, interleukin-6; IL-10, interleukin-10; n, number of observations.

Supplementary Figure S1. Panel of line graphs showing IL-6 levels and total seizures changes over time for individual patient. Stata version 16.1 (StataCorp, College Station, Texas, USA) was used to prepare line graphs and Excel (Microsoft Excel for Microsoft 365 MSO) was used to draw Figures S1-S3.

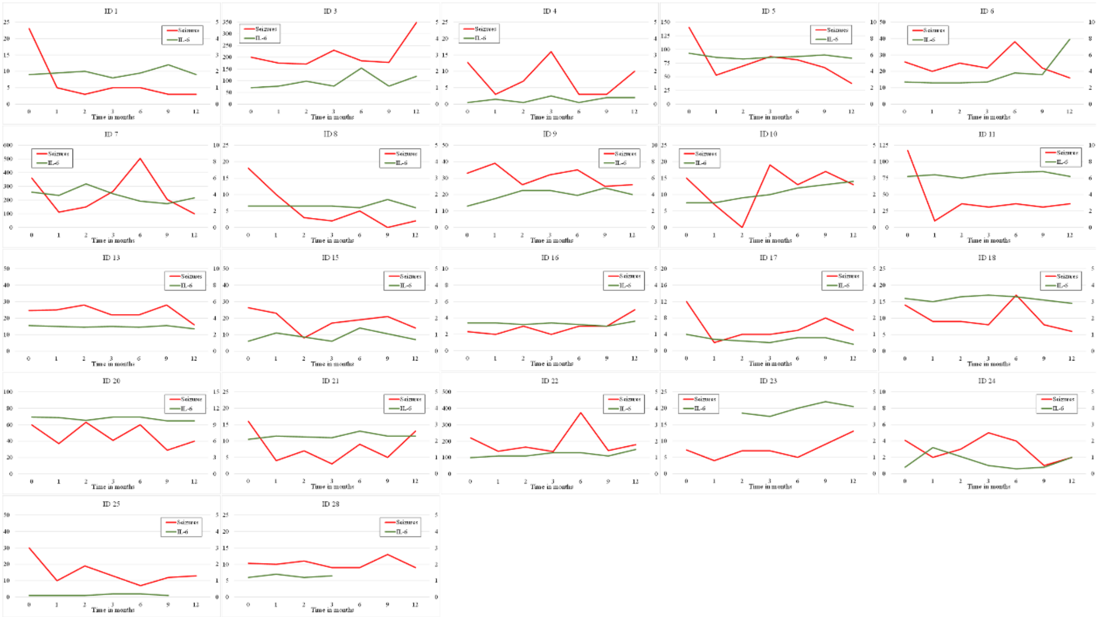

Supplementary Figure S2. Panel of line graphs showing IL-10 and total seizures changes over time for individual patient

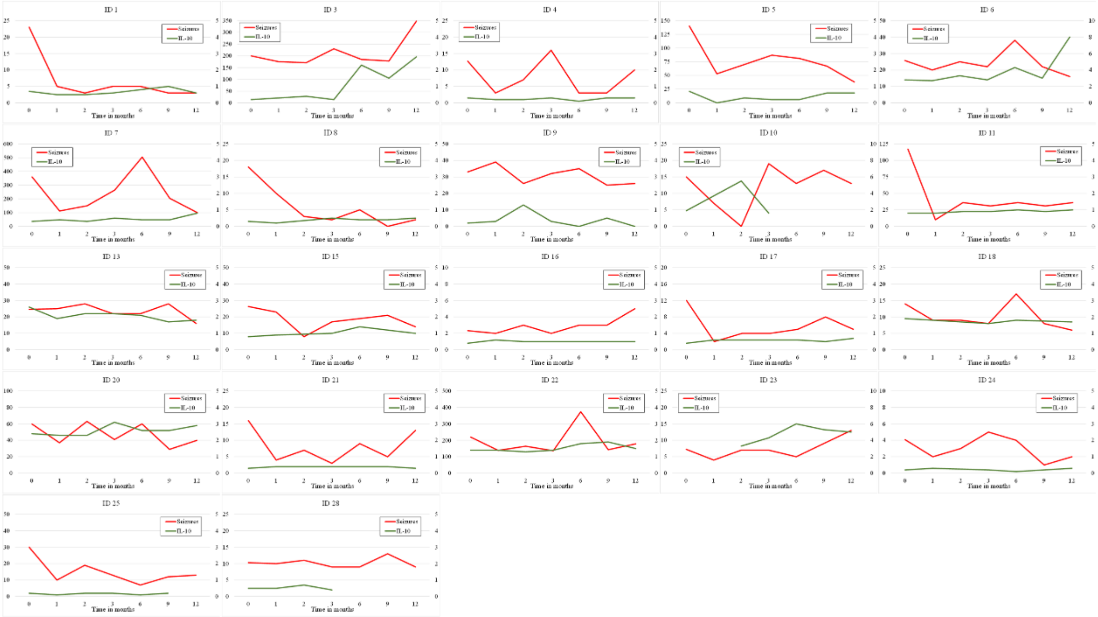

Supplementary Figure S3. Panel of line graphs showing IL-6/IL-10 ratio and total seizures changes over time following ANT-DBS treatment for individual patient.

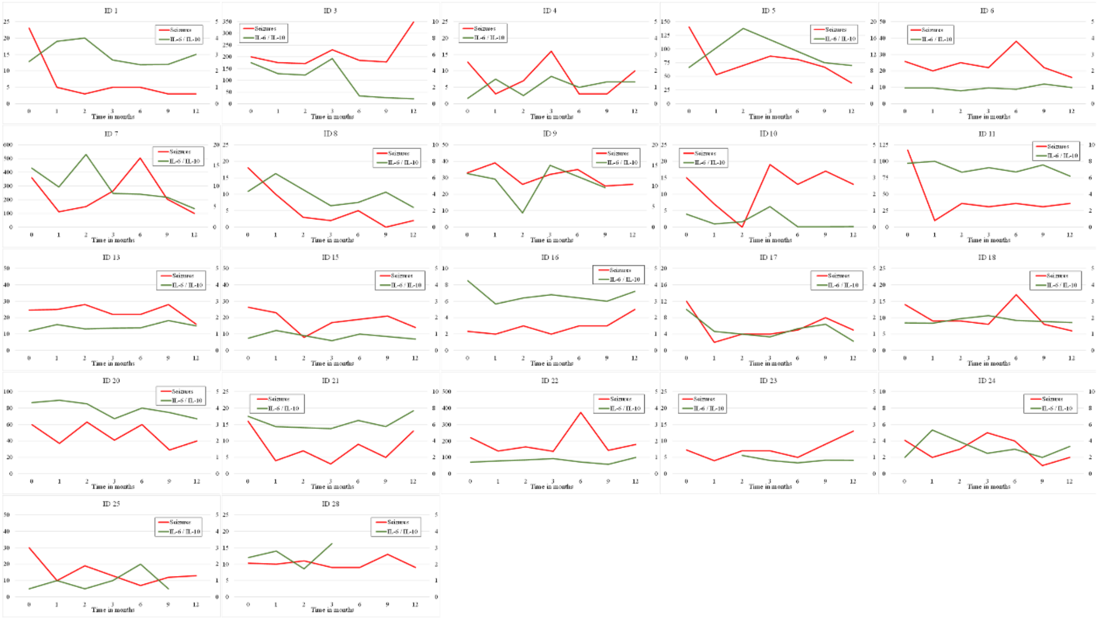

Supplement: Supplementary file 1 — Supplementary Information. [file 41598_2021_93265_MOESM1_ESM.pdf]
